# Differences by sex and gender in the association between minority stress and alcohol use among sexual and gender minority youth: A daily diary study

**DOI:** 10.1016/j.socscimed.2021.114679

**Published:** 2021-12-24

**Authors:** W.J. Kiekens, L. Baams, R. Veenstra

**Affiliations:** a Department of Sociology/Interuniversity Center for Social Science, Theory and Methodology (ICS), University of Groningen, the Netherlands; b Department of Pedagogy and Educational Sciences, University of Groningen, the Netherlands

**Keywords:** Diary study, Alcohol use, Sexual and gender minority, Youth, LGBT, Minority stress

## Abstract

**Rationale:**

Sexual and gender minority (SGM) youth consume more alcohol than their heterosexual, cisgender peers. The experience of minority stress is theorized to explain these disparities. Research often neglects the day-to-day variability in minority stress that SGM youth encounter and whether alcohol use is associated with daily experiences of minority stress. Further, there is heterogeneity in alcohol use among SGM youth. Sex assigned at birth and gender identity could potentially explain this heterogeneity.

**Objective:**

Therefore, this study aimed to examine whether daily experiences of minority stress were associated with daily alcohol use among SGM youth and how these associations differed by sex assigned at birth and gender identity.

**Methods:**

A 14-day daily diary study was conducted among 393 Dutch SGM youth (*M* age = 18.36 *SD* = 2.65).

**Results:**

Results showed few significant associations between both mean levels of minority stress and daily experiences with minority stress with alcohol use. However, higher mean levels of prejudice events were associated with higher odds of daily alcohol use (*OR* = 7.01, 95% *CI:* 1.20–40.89). Daily experiences with identity concealment were associated with lower odds of daily alcohol use for males (*OR* = 0.72, 95% *CI:* 0.60–0.86), but not for females (*OR* = 1.11, 95% *CI:* 0.93–1.32). Further, for cisgender youth, daily experiences with prejudice events were associated with higher odds of alcohol use (*OR* = 1.99, 95% *CI:* 1.05–3.78), but this was not the case for gender minority youth (*OR* = 0.42, 95% *CI:* 0.15–1.18).

**Conclusions:**

The findings showed few significant associations between minority stressors and alcohol use, but daily experiences of concealment and prejudice events were associated with daily alcohol use and these associations varied by sex assigned at birth and gender identity, respectively.

## 1 Introduction

Alcohol use is a serious health problem for adolescents and young adults (Hall et al., 2016). Certain subpopulations of youth have a higher risk of alcohol consumption, such as sexual and gender minority (SGM) youth (Marshal et al., 2008; Mereish, 2019). A meta-analysis showed that sexual minority adolescents are 2.55 times more likely to consume alcohol than their heterosexual peers (Marshal et al., 2008). Data from the California Healthy Kids Survey showed that transgender adolescents reported higher past month alcohol use than cisgender adolescents (Coulter et al., 2018). Most of the existing research on sexual and gender identity disparities in alcohol use is conducted in the United States, but similar alcohol use disparities are found in other countries, such as the Netherlands (Kuyper, 2015).

Higher alcohol use rates among SGM youth are often explained by their experiences with minority stress. Minority stress is understood as stress related to one's sexual or gender identity that SGM people experience additional to general stressors (Meyer, 2003; Testa et al., 2015). Despite giving valuable insights into alcohol use among SGM youth, existing research often neglects the day-to-day variability in experiences with minority stress and associated daily alcohol use. Research has called for such a within-person approach in studying alcohol use among SGM youth (Watson et al., 2019) and how minority stressors might affect alcohol use (Livingston, 2017). This study aimed to answer this call by investigating whether daily minority stress experiences relate to daily alcohol use among SGM youth.

### 1.1 Minority stress and alcohol use

The minority stress framework and its extensions (Hatzenbuehler, 2009; Meyer, 2003; Testa et al., 2015) provide a potential explanation for disparities in alcohol use among SGM youth. Four minority stressors, ranging on a continuum from distal to proximal, are distinguished: (1) acute prejudice events, for example, being verbally assaulted or physically victimized; (2) the expectation of prejudice events and the vigilance this brings; (3) the concealment of one's minority identity; and (4) internalized stigma, which refers to the internalization of society's negative attitudes toward a minority identity. Together, these experiences of minority stress are thought to predict alcohol use among SGM youth (Goldbach et al., 2014; Mereish, 2019).

It has been proposed that associations between minority stress and alcohol use can be accounted for by coping/emotion regulation processes (Hatzenbuehler, 2009). Following the transactional theory of stress coping (Lazarus and Folkman, 1984), alcohol can be used as a coping mechanism to (minority) stress. For example, when a situation is appraised as stressful, and efforts are needed to manage or resolve the situation, people may engage in certain coping actions, including alcohol use (Biggs et al., 2017), thereby potentially reducing negative and increasing positive affect (Wills and Hirky, 1996). Indeed, in a study among same-sex attracted Dutch youth, *drinking to cope* explained the association between same-sex attraction and drinking on weekdays (Bos et al., 2016). Further, in a sample of sexual minority women, *drinking to cope with negative feelings* was related to heavy episodic drinking (Fish and Hughes, 2018). In sum, SGM youth may use alcohol to cope with minority stress or the negative feelings minority stress may elicit.

Researchers provide evidence for an association between minority stress and alcohol use among SGM youth. For example, sexual identity victimization was associated with alcohol use and binge drinking among sexual minority adolescents (Coulter et al., 2018). Further, sexual identity-related micro-aggressions and experiences with victimization were associated with lifetime alcohol use and alcohol problems among men who have sex with men (Dyar et al., 2019). Similarly, bullying-victimization explained disparities in alcohol use between gender minority and cisgender youth (Reisner et al., 2015). Further, in a sample of LGB emerging adults, low levels of concealment and higher levels of outness were related to harmful alcohol use, and this association was explained by heterosexist experiences (Villarreal et al., 2020). Last, research has shown that internalized stigma was related to excessive alcohol use among transgender men but not transgender women (Gonzalez et al., 2017). Taken together, minority stressors relate to alcohol use among SGM youth, but how this operates on a daily level is not yet understood.

### 1.2 A daily diary approach

Although research has been informative in understanding the links between minority stress and alcohol use, we identify three drawbacks of research on this association among SGM youth. First, research rarely tests multiple minority stressors to explain alcohol use simultaneously. That is, research either assesses single minority stressors, for example, only prejudice events (Coulter et al., 2018), or research uses a proxy of minority stress processes, such as assessing general victimization as an indicator for prejudice events (Kiekens et al., 2020). This limits our understanding of how multiple minority stressors together relate to SGM youth's alcohol use. Second, studies often ask youth about past experiences with minority stress and past alcohol use. A downside of this approach is that youth can experience difficulties reconstructing past experiences (Larson and Csikszentmihalyi, 2014), especially concerning stressful experiences with discrimination (Sechrist et al., 1998). This may result in bias reporting minority stressors and alcohol use. Third, existing research has focused on between-person differences when studying the association between minority stress *and* alcohol use. Such a focus on individual differences neglects the day-to-day within-person variability in minority stress and whether alcohol is used in response to daily experiences with minority stress. Studying within-person variability in minority stress and alcohol use provides a more comprehensive understanding of how minority stressors are associated with alcohol use among SGM youth.

With a daily diary approach, we overcome these shortcomings because youth give a daily report on their experiences of minority stress and alcohol use. Some research has used a daily diary approach to examine links between daily experiences of minority stress and health among SGM people. For example, it was found that on days when sexual minority adults had a heterosexist experience, feelings of anger increased (Mohr, 2016). Further, sexual minority adults reported a decrease in positive affect on days with higher levels of internalized stigma or expected rejection (Mohr and Sarno, 2016) or days with higher rates of concealment (Beals et al., 2009). Daily discrimination was also associated with momentary anxious and depressed mood among SGM people (Livingston et al., 2020), and daily minority stress was related to lower positive and higher negative affect among sexual minority men (Eldahan et al., 2017). Among gender minority people, daily distal minority stressors predicted daily drug use (Wolford-Clevenger et al., 2021). Finally, daily sexual and gender identity-related discrimination was linked to daily nicotine and substance use (Livingston et al., 2017). In sum, a daily diary approach is a suitable and feasible approach to study the associations between daily experiences with minority stress and alcohol use.

### 1.3 Sex, and gender identity-group differences

Although SGM youth evidence higher alcohol use rates than their heterosexual and cisgender peers (Marshal et al., 2008; Mereish, 2019), there is heterogeneity in alcohol use among SGM youth. Sex assigned at birth is a source of this heterogeneity (Marshal et al., 2008; Mereish, 2019). Especially sexual minority women report higher alcohol use in representative US studies than heterosexual youth (Johns et al., 2018; Kann et al., 2016). Research findings of group-differences among sexual minority youth are mixed. For example, in a national non-probability sample, sexual minority adolescents assigned male at birth reported higher alcohol use than assigned female sexual minority adolescents (Watson et al., 2020), whereas research using a national representative sample pointed to the opposite (Kann et al., 2016). Gender identity is an additional source of heterogeneity in alcohol use. For example, transgender youth reported higher odds of ever using alcohol than cisgender boys in a national study (Reisner et al., 2015) and reported higher past month alcohol use than cisgender adolescents in a study among Californian high school students (Coulter et al., 2018). Further, transgender adolescents reported higher lifetime alcohol use than cisgender sexual minority adolescents, whereas nonbinary and genderqueer adolescents reported lower recent alcohol use than cisgender sexual minority adolescents (Watson et al., 2020). Thus, sex assigned at birth and gender identity are potential sources of heterogeneity in alcohol use among SGM youth. However, few studies assessed factors that may explain heterogeneity in alcohol use among SGM youth, and research has called to do so (Mereish, 2019).

A potential explanation for group-differences in alcohol use among SGM youth is level and response to minority stress. Experimental research suggests that high exposure to minority stress may result in a blunted stress response among sexual minority young adults (Hatzenbuehler and McLaughlin, 2014; Mereish and Miranda, 2021), and such a stress response may drive higher alcohol use or addiction (Sinha, 2008). Thus, SGM youth with a relatively high exposure to minority stress may have a stronger stress response than SGM youth with a relatively low exposure to minority stress, which could lead to differences in alcohol use. Considering that sexual minority men are more often exposed to minority stressors and negative attitudes than sexual minority women (Katz-Wise and Hyde, 2012; Kite and Whitley Jr, 2003; Pew Research Center, 2013), sex assigned at birth may moderate the association between minority stress and alcohol use among SGM youth. Similarly, gender minority people experience the highest levels of prejudice and stigma among SGM people (Martín-Castillo et al., 2020; Su et al., 2016). Thus, gender identity may moderate the association between minority stress and alcohol use among SGM youth.

### 1.4 The present study

Our aim was to examine whether daily experiences of minority stress were related to daily alcohol use among SGM youth and how these associations differed by sex assigned at birth and gender identity. We expected that daily experiences of prejudice events, expectations of rejection, the concealment of one's identity, and internalized stigma would independently predict daily alcohol use. Further, to explain heterogeneity in alcohol use, we explored whether sex assigned at birth and gender identity moderated the association between daily minority stressors and daily alcohol use.

## 2 Methods

### 2.1 Design and procedure

Participants were recruited through paid advertisements on Facebook and Instagram in 2019. Advertisements were targeted at SGM youth who were 16–25 years old, lived in the Netherlands, spoke Dutch, and had sexual and gender minority-related interests (e.g., Gay Pride Parade). On the advertisement picture, it was noted that the study focused on “LGBTQ + people and people who were (also) attracted to people of the same sex”, that participants would be asked to complete a 14-day diary, and that they would be compensated with a €25 gift card. Youth interested in the study were directed to a website with more information on the study's procedure. Here it was explained that for every completed daily survey, participants would receive €1.79, which could amount to a total of €25 in gift cards (14 days at circa €1.79 per day = €25). This approach was deemed as the best option for compensation in diary studies (Hall and Nishina, 2019). Interested participants were directed to the informed consent form. After that, participants were asked to fill in their email addresses to receive their gift card and their phone number to receive texts with a link to the daily surveys. On the first study day, participants completed a baseline survey and the first daily survey. For the following thirteen days, participants received a text message at 5 p.m. with a link to a new daily survey and a reminder text message at 8 p.m. All surveys were constructed in Qualtrics. The Ethics Committee of the Pedagogy and Educational Sciences Department of the University of Groningen approved the study’s procedure.

The survey was developed in collaboration with three sexual and gender minority youth, one secondary education teacher, and two healthcare professionals. During three focus group meetings, youth, teachers, and healthcare professionals were asked to comment on the survey content, ensure inclusiveness of the response options, and comment on the clarity and relevance of our measures.

### 2.2 Participants

The initial sample comprised of *N* = 409 participants. Participants who completed the consent form but did not complete a question (*n* = 13) or a daily survey (*n* = 3) were removed from the data. This resulted in an analytic sample of *N* = 393 who completed at least one daily survey. Most participants identified as cisgender men (32.3%) or women (46.3%) and smaller groups identified as transgender men (5.6%), transgender women (0.8%), non-binary (3.8%), genderqueer (2.0%), genderfluid (1.0%), a different gender identity (0.8%), or did not know their gender identity (7.4%). The sample was diverse in terms of sexual identity, with participants identifying as lesbian/gay (43.7%), bisexual (29.5%), queer (7.4%), pansexual (9.9%), heterosexual, (1.3%) do not know (5.3%), and a different minority sexual identity (2.8%, e.g., asexual, omnisexual). The mean age was 18.36 (*SD* = 2.65), and a total of 13.5% of participants did not identify as Dutch. Of all participants, 80.4% were in secondary or higher education. Of those, 41.1% were in high school, 23.7% in vocational education, 33.9% attended (applied) university, and 1.3% had a different education. Of those not in school, the majority (56.6%) had only a high school diploma, whereas smaller groups had a vocational education diploma (11.8%), an (applied) university degree (28.9%), or a different degree (2.6%). In total, 75.3% of the participants lived with their parents.

Of all participants, 57.0% fell under the legal drinking age of 18 years old. Nevertheless, we expected that this group would have at least some experience with alcohol use as the findings of recent Dutch research indicated that 71.2% of 16 years old reported ever drinking alcohol, 53.3% drank alcohol in the past month, and 34.2% reported being drunk in the past month (Rombouts et al., 2020). Thus, despite the young age of most participants, we expected that many would have consumed alcohol at least once.

### 2.3 Measures

#### Daily alcohol use

On the first day, daily alcohol use was assessed by asking about alcohol consumption in the past 24 h. For the remaining thirteen days, the item read “Did you consume alcohol since completing the previous daily survey?”. Response options were 0 = *No* and 1 = *Yes*.

#### Daily prejudice events

Daily prejudice events were assessed with the following item “Since completing the previous daily survey (on the first day: 'In the past 24 h'), did you have a negative experience related to your sexual orientation or gender identity? For example, annoying jokes, inappropriate questions, being excluded, or being called names.” This item was adapted from a daily diary study on heterosexism (Mohr and Sarno, 2016). Answer categories were 0 = *No* and 1 = *Yes*. Participants' daily prejudiced events were averaged across all completed days to assess prejudiced events at the person level. These scores can be interpreted as the proportion of days featuring prejudice events.

#### Daily expectations of rejection

Daily expectations of rejection were assessed with the item “Since completing the previous daily survey (on the first day: ‘In the past 24 h’), I was afraid something negative would happen related to my sexual orientation or gender identity. For example, annoying jokes, inappropriate questions, being excluded, or being called names.” Answer categories ranged from 1 = *Completely disagree* to 5 = *Completely agree.* Participants' daily expectations of rejection scores were averaged across all completed days to assess expectations of rejection at the person level. These scores can be interpreted as participants' mean scores of expectations of rejection across all completed days.

#### Daily concealment

Daily concealment was assessed with the following item “Since completing the previous daily survey (on the first day: 'In the past 24 h'), I have done something to conceal my sexual identity or gender identity. Think about examples, such as not holding hands with your partner, dressing differently, not telling others something about yourself.” Answer categories ranged from 1 = *Completely disagree* to 5 = *Completely agree.* Participants' daily concealment scores were averaged across all completed days to assess concealment at the person level. These scores can be interpreted as participants' mean score of concealment across all completed days.

#### Daily internalized stigma

Two items assessed daily internalized stigma. Participants were first asked, “Today I was insecure about my sexual orientation or gender identity”. Second, participants were asked, “Today I wished I was heterosexual or cisgender” (Mohr and Kendra, 2011). Answer categories ranged from 1 = *Completely disagree* to 5 = *Completely agree,* and the mean response of these two items was used in the analyses. Participants' daily internalized stigma scores were averaged across all completed days to assess internalized stigma at the person level. These scores can be interpreted as participants' mean score of internalized stigma across all completed days.

#### Sexual identity

In the baseline survey, sexual identity was assessed with the following item: “How would you describe your sexual identity?” with answer options: 1 = *Lesbian,* 2 = *Gay,* 3 = *Bisexual,* 4 = *Queer,* 5 = *Pansexual,* 6 = *Heterosexual,* 7 = *I don't know,* and 8 = *Other, namely.* Sexual identity was recoded as 1 = *Lesbian/Gay,* 2 = *Bisexual,* 3 = *Queer/Pansexual/Heterosexual/I don't know/Other, namely* for our analyses. *Lesbian/Gay* was used as the reference group as this was the largest group.

#### Sex assigned at birth and gender identity

Sex assigned at birth was assessed with the following item: “What is the sex you were assigned at birth?” with answer options 1 = *Male,* 2 = *Female,* and 3 = *Other, namely.* There were no participants that answered 3 = *Other, namely.* Responses were recoded such that 0 = *Male* and 1 = *Female.* In the baseline survey, gender identity was assessed with the following item “How would you describe your gender identity?” with answer options 1 = *Man,* 2 = *Woman,* 3 = *Transgender man,* 4 = *Transgender woman,* 5 = *Non-binary,* 6 = *Genderqueer,* 7 = *Genderfluid,* 8 = *Don’t know,* and 9 = *Other, namely.* Gender identity was recoded as 0 = *Cisgender* when participants reported their sex assigned at birth as *Female* or *Male,* and gender identity as *Woman* or *Man,* respectively. If participants reported another gender identity or their gender identity did not align with their sex assigned at birth, gender identity was recoded as 1 = *Gender minority.* For the purpose of these analyses, the subgroups for non-binary, genderqueer, and genderfluid youth were too small to compare separately.

#### Day of Study

A variable was created to represent the day of the survey, coded with 1 = the first day, 2 = the second day, and so on. This variable was created to control for the effects of repeated measurements.

#### Weekend day

Because drinking patterns differ during weekdays and weekends, a variable was created indicating whether a diary entry reflected a *weekday* (= 0) or *weekend* (= 1). As Thursday is a typical day to drink among Dutch students, diary entries from Friday (i.e., reflected on Thursday), Saturday (i.e., reflected on Friday), and Sunday (i.e., reflected on Saturday) were coded as *weekend.*

### 2.4 Analytic strategy

Because observations were nested within individuals and because the outcome variable was binary, multilevel logistic regression analyses were used to analyze associations between minority stress and alcohol use at both the between- and within-person level (Hoffman, 2015), using Mplus version 8.3 (Muthén & Muthén, 1998–2017). Predictor variables at the within-person level were daily prejudice events, daily expectations of rejection, daily concealment, daily internalized stigma, day of study, and weekend day. Predictor variables at the between-person level were person-level prejudice events, person-level expectations of rejection, person-level concealment, person-level internalized stigma, sexual identity, sex assigned at birth, and gender identity. Daily prejudice events, expectations of rejection, concealment, and internalized stigma were centered at each person’s mean, as recommended when studying cross-level interactions (Enders and Tofighi, 2007). Person-level prejudice events, expectations of rejection, concealment, and internalized homophobia were centered at the overall mean.

Multilevel logistic regression analyses were conducted in four steps. First, Model 0 included no predictor variables, also referred to as the empty model, in which the intraclass correlation (ICC) can be calculated (Sommet and Morselli, 2017). In Model 1, control variables (i.e., day of study, weekend day, sex assigned at birth, gender identity, and sexual identity) and all daily and person-level minority stressors were added in one single model. In all models, the slopes of the daily minority stress variables were allowed to vary randomly, representing each participant's unique association between daily minority stressors and alcohol use. In Model 2, a cross-level interaction was added between daily minority stressors and sex assigned at birth to assess sex-based differences in the association between daily minority stressors and alcohol use. Last, Model 3 presents the cross-level interaction between daily minority stressors and gender identity to assess gender identity-based differences in the association between daily minority stressors and alcohol use. For significant interaction terms, simple slopes were tested (Stride et al., 2015).

There was no missing data for any of the between person-level variables. At the within person-level, daily prejudice events, daily expectations of rejection, daily concealment, daily internalized stigma, and alcohol use had missing values (see Table 1). Missing data analyses suggested that data were missing completely at random (MCAR) and that multiple imputation would be a sufficient procedure to take into account this type of missingness (Schafer and Graham, 2002). The IMPUTATION option in Mplus was used to impute the data. As per the study design, participants were allowed to skip a diary day. Therefore, skipped days were not imputed. Analyses using imputed data and analyses using listwise deletion yielded similar results.

This study also assessed daily tobacco and marijuana use. Because a relatively low number of participants used these substances during the two weeks of the study and variability among users was low, models showed signs of misspecifications. Therefore, we only include models for alcohol use in our study.

## 3 Results

### 3.1 Descriptive results

The number of participants that completed daily surveys across the 14 days of the study declined, with *N* = 393 participants starting on the first day and *n* = 324 that completed day 14 (82.4%, see Table 1). On average, participants completed 12.01 diaries (*SD* = 3.51). Table 1 also presents descriptive statistics for all key variables. Across the 14 days of the study, participants drinking on a certain day ranged from 10.9% to 20.8%. In total, 58.5% of the participants drank alcohol.

### 3.2 Multilevel analyses

Table 2 presents findings from the multilevel analyses for daily alcohol use. Only significant findings are discussed here. In Model 0 (not presented in Table 2), the ICC was obtained: differences between people explained 48% of the likelihood of alcohol use, 30% in daily prejudice events, 17% in daily expectations of rejection, 22% in daily concealment, and 18% in daily internalized stigma. Model 1 (see Table 2) indicates that higher scores on person-level prejudice events were associated with higher odds of daily alcohol use (*OR* = 7.01, 95% *CI:* 1.20–40.89]). Results showed no significant associations between alcohol use and any of the other person-level minority stressors or for daily minority stressors. Further, youth assigned female at birth had lower odds of daily alcohol use than youth assigned male at birth (*OR* = 0.41, 95% *CI:* 0.23–0.71). We found no significant associations between sexual or gender identity and alcohol use.

In Model 2, we added cross-level interaction effects between daily minority stressors and sex assigned at birth. A deviance test indicated that adding the cross-level interaction terms resulted in a significant model improvement compared to the model without cross-level interaction terms (i.e., Model 1) (χ^2^(4) = 11.67, *p* = .02). Only the cross-level interaction effect of sex assigned at birth and daily concealment was significant (*OR* = 1.54, 95% *CI:* 1.24–1.92). Simple slope analyses showed that for assigned males daily concealment was associated with lower odds of daily alcohol use (*OR* = 0.72, 95% *CI:* 0.60–0.86) and for assigned females, no significant association between daily concealment and daily alcohol use was found (*OR* = 1.11, 95% *CI:* 0.93–1.32).

In Model 3, the cross-level interaction effects between daily minority stressors and gender identity were estimated. The interaction terms resulted in a significant model improvement compared to Model 1 (χ^2^(4) = 13.48, *p* < .01). We only found a significant cross-level interaction effect between gender identity and daily prejudice events (*OR* = 0.21, 95% *CI:* 0.08–0.58). Simple slope analyses showed that for cisgender youth daily experiences of prejudice events were associated with higher odds of daily alcohol use (*OR* = 1.99, 95% *CI:* 1.05–3.78), whereas for gender minority youth, no significant association between daily prejudice events and daily alcohol use was found (*OR* = 0.42, 95% *CI:* 0.15–1.18).

### 3.3 Robustness check

Several robustness checks were conducted. First, the classical false discovery rate method (FDR) was used to correct for multiple testing (Benjamini and Hochberg, 1995). The *p* values of all significance tests in a specific model were ordered from smallest to largest and a result was statistically significant if for the *i*’th ordered *p-value p(i)* ≤ α × *i*/*m*, where α was set at .05, *i* is the ranking in the order of *p* values, and *m* is the total number of tests conducted in a specific model. All findings were held when using the FDR method.

Second, models were rerun separately for each minority stressor to assess daily minority stressors' independent associations with daily alcohol use (see Tables S1A-D in the online supplementary). Two differences in results emerged. First, person-level prejudice events were only marginally significantly associated with alcohol use in Model 1 (*OR* = 4.09, 95% *CI:* 0.90–18.49). Second, simple slope analyses showed that for cisgender youth, daily prejudice events were only marginally significantly associated with lower odds of daily alcohol use (*OR* = 1.78, 95% *CI:* 0.97–3.26). Thus, running models separately for each minority stressor did not yield new significant associations, which indicated that no effects were suppressed by modeling all daily minority stressors together.

Third, models were rerun omitting day 1 responses because a different time frame was used compared with the other days (see online supplementary Table S2). Overall, three differences in results emerged. First, the interaction between daily concealment and sex assigned at birth resulted only in a marginal significant model improvement in Model 2 (*χ*^2^ (4) = 8.76, *p* = .07). Second, simple slope analyses indicated no differences in the association between daily prejudice events and alcohol use by gender identity in Model 3, although directions of the simple slope effects were similar compared with models including day 1. Third, simple slope analyses showed that for gender minority youth daily expectations of rejection were associated with higher odds of daily alcohol use in Model 3 (*OR* = 1.48, 95% *CI:* 1.03–2.13), but no significant association for cisgender youth was found (*OR* = 1.02, 95% *CI:* 0.85–1.22). Thus, the findings with and without day 1 were fairly similar, especially considering the lower statistical power of these analyses.

Last, we estimated-cross lagged models to examine bidirectional associations between daily minority stress and next-day daily alcohol use (see online supplementary Table S3A-D). We only found a significant negative cross-lagged association of daily internalized stigma on daily alcohol use (b = –0.04, *se* = 0.02, *p* = .03). Multi-group models for sex assigned at birth and gender identity were estimated as well and provided evidence for cisgender participants for the same negative cross-lagged association of daily internalized stigma on daily alcohol use (*b* = –0.06, *se* = 0.02, *p* = .02).

## 4 Discussion

We aimed to examine whether daily experiences of prejudice events, expectations of rejection, the concealment of one's identity, and internalized stigma were related to daily alcohol use among SGM youth and how these associations differed by sex assigned at birth and gender identity.

Few associations between both mean-level minority stressors and daily minority stressors with daily alcohol use were found. However, we found that higher mean levels of prejudice events were associated with higher odds of alcohol use, although this association was marginally significant in a robustness check where we ran models for each minority stressor separately. Further, contrary to our expectations, daily experiences with identity concealment were associated with lower odds of daily alcohol use for males but were not associated with females' daily alcohol use. This effect resulted only in a marginal model improvement in a robustness check where we omitted day 1 responses. Although concealing one's identity is conceptualized as a minority stressor (Meyer, 2003), it may also serve as a protective factor. That is, individuals who conceal their minority identity may be less exposed to minority stressors such as prejudice events (Pasek et al., 2017), which could result in lower levels of *drinking to cope.* Nonetheless, one should be careful framing concealment as a protective factor as it has been theorized to negatively affect mental health (Pachankis, 2007). With our study we cannot infer what other effects concealment may have had. Alternatively, alcohol use among SGM people might also be explained by social norms around alcohol use in the SGM community (Green and Feinstein, 2012). It is possible that SGM youth who regularly conceal their sexual or gender identity were socialized less with SGM youth, and thus did not traverse social contexts with these social norms, resulting in a negative association between concealment and alcohol use. Last, for cisgender youth, daily experiences of prejudice events were associated with higher odds of daily alcohol use, but not for gender minority youth, although this association became marginally significant in a robustness check where we ran models for each minority stressor separately and disappeared in a robustness check where we omitted day 1 responses. The finding for cisgender sexual minority youth is in line with previous research (Coulter et al., 2018; Dyar et al., 2019, 2020) and extends previous findings to the daily level. It shows that prejudiced events may play a role in alcohol consumption during adolescence and perhaps even in (young) adulthood (Evans-Polce et al., 2020). Not finding this association for gender minority youth may be explained by the small sample of gender minority youth in our study and should be interpreted with caution. Together, few associations between minority stressors and alcohol use were identified, but we found some evidence that daily experiences with minority stressors, more specifically daily concealment and prejudice events, were associated with alcohol use on a daily level for male sexual minority youth and cisgender sexual minority youth, respectively.

Contrary to our expectations, we did not find associations between daily expectations of rejection and internalized stigma with alcohol use. Research has noted that associations between proximal minority stressors and health outcomes are small (Brubaker et al., 2009; Newcomb and Mustanski, 2010; Pachankis et al., 2020). Further, in the cross-lagged models, we found that internalized stigma was associated with *lower* next-day alcohol use, indicating that internalized stigma may function similarly to concealment. However, considering that a priori power analyses pointed to a power of 0.84 to detect medium to large effect sizes (Bolger et al., 2011), it could be that the association between these proximal minority stressors with daily alcohol use was too small to detect with our sample size. Alternatively, our findings could also suggest that expectations of rejection and internalized stigma are not associated with alcohol use on a daily level. That is, expectations of rejection and internalized stigma may not contribute to alcohol use momentarily, but only when they accumulate and become chronic or persistent (Meyer, 2003). Future diary studies are needed to substantiate these findings.

Last, research suggests that bisexual youth report the highest rates of alcohol use among sexual minority youth (Marshal et al., 2008; Mereish, 2019) which we did not find. Similarly, we found no differences in alcohol use between cisgender and gender minority youth, despite previous research among SGM youth pointing to such differences (Watson et al., 2020). A possible reason for these null findings could be that sexual orientation and gender minority subgroups were too small to compare.

### 4.1 Strengths and limitations

This is one of the first daily diary studies among SGM youth to assess the association between daily experiences of minority stress with alcohol use among SGM youth. We found some indications that daily minority stressors may raise the risk of maladaptive coping strategies such as alcohol consumption and emphasize the burden of these daily experiences with minority stress for SGM youth.

Our findings should be interpreted in light of some limitations. First, the findings may be difficult to generalize because advertisements on Facebook and Instagram only targeted people with sexual and gender identity-related interests. This may exclude youth who are unsure of their identity or who fear being outed to friends and family when they would show these interests on their social media profiles. Future diary studies, perhaps using different sampling strategies, are needed to substantiate the present study's findings. Second, we only considered whether or not youth had consumed alcohol on a given day and could only make inferences on the odds of daily alcohol use. Therefore, no statements can be made about the relationship between minority stressors and the severity of daily alcohol use, which is important as the amount of alcohol consumed is relevant for associated health risks (Siqueira and Smith, 2015). Third, although using a daily diary approach recall bias was reduced, social desirability bias could have impacted our findings, especially concerning alcohol use reports (Krumpal, 2013). Fourth, despite the longitudinal design, we cannot make any inferences about the causality of the associations found in the present study, especially considering the marginal fit of some of the cross-lagged models. For example, it is possible that daily prejudice events and alcohol use were related because both occurred in the same contexts (e.g., bars or clubs). Last, perceived alcohol use norms may be a potential confounder because previous findings indicate more permissive alcohol use norms in SGM communities (Green and Feinstein, 2012) and a link with sexual minority people's alcohol use (Boyle et al., 2020).

Meta-analyses reported small associations between minority stressors and health outcomes (Newcomb and Mustanski, 2010; Pachankis et al., 2020). To better understand the risk of alcohol consumption among SGM youth, future research should focus on other drivers of alcohol use besides minority stress, for example rejection sensitivity (Feinstein, 2020). It may be that youth high in rejection sensitivity react to perceived ambiguous events with alcohol use (Baams et al., 2020), although research among sexual minority men did not find a direct effect of rejection sensitivity on alcohol use (Pachankis et al., 2014).

### 4.2 Conclusion

This study examined the association between daily minority stressors and daily alcohol use among Dutch SGM youth. Few significant associations between minority stressors and alcohol use were identified. Nevertheless, daily experiences of concealment and prejudice events were associated with daily alcohol use and these associations varied by sex assigned at birth and gender identity, respectively. This research answered calls to take a within-person approach to study alcohol use among sexual and gender minority youth (Livingston, 2017; Watson et al., 2019) and provided evidence of how minority stress processes affect alcohol use on a daily level.

## Supplementary Material

Supplementary data to this article can be found online at https://doi.org/10.1016/j.socscimed.2021.114679.

Supplementary Material

## Figures and Tables

**Table 1:** Descriptive statistics for key variables by day.

Day	*N* (%)	Prejudice events		Expectations of rejection			Concealment			Internalized homophobia			Alcohol use	
		Yes (%)	*N* missing (%)	*M*	*SD*	*N* missing (%)	*M*	*SD*	*N* missing (%)	*M*	*SD*	*N* missing (%)	Yes (%)	*N* missing (%)
1	393 (100.0)	46 (11.7)	0 (0.0)	2.25	1.33	0 (0.0)	2.39	1.46	0 (0.0)	1.87	0.96	0 (0.0)	82 (20.8)	0 (0.0)
2	365 (92.9)	41 (10.4)	1 (0.3)	1.98	1.18	2 (0.5)	2.13	1.34	2 (0.5)	2.00	1.23	1 (0.3)	58 (15.9)	0 (0.0)
3	350 (89.1)	30 (7.6)	0 (0.0)	1.99	1.22	5 (1.4)	2.08	1.34	3 (0.9)	1.82	1.11	0 (0.0)	61 (15.4)	1 (0.3)
4	341 (86.8)	25 (6.4)	3 (0.9)	1.75	1.09	6 (1.8)	1.87	1.21	6 (1.8)	1.86	1.18	4 (1.2)	49 (12.4)	1 (0.3)
5	337 (85.8)	34 (8.7)	0 (0.0)	1.89	1.17	4 (1.2)	1.87	1.22	2 (0.6)	1.81	1.15	1 (0.3)	43 (10.9)	0 (0.0)
6	340 (86.5)	29 (7.4)	1 (0.3)	1.81	1.17	3 (0.9)	1.95	1.27	2 (0.6)	1.84	1.18	2 (0.6)	65 (16.4)	0 (0.0)
7	332 (84.5)	22 (5.6)	2 (0.6)	1.90	1.21	4 (1.2)	1.96	1.29	2 (0.6)	1.80	1.19	2 (0.6)	64 (16.2)	1 (0.3)
8	329 (83.7)	27 (6.9)	0 (0.0)	1.85	1.17	3 (0.9)	1.90	1.24	2 (0.6)	1.81	1.17	1 (0.3)	73 (18.4)	1 (0.0)
9	331 (84.2)	14 (3.6)	1 (0.3)	1.80	1.12	7 (2.1)	1.84	1.20	3 (0.9)	1.70	1.10	2 (0.6)	54 (13.6)	0 (0.0)
10	326 (83.0)	20 (5.1)	2 (0.6)	1.81	1.14	5 (1.5)	1.85	1.21	5 (1.5)	1.79	1.17	3 (0.9)	52 (13.1)	1 (0.3)
11	324 (82.4)	24 (6.1)	0 (0.0)	1.77	1.13	4 (1.2)	1.83	1.23	3 (0.9)	1.80	1.15	0 (0.0)	43 (10.9)	0 (0.0)
12	318 (80.9)	21 (5.3)	1 (0.3)	1.82	1.15	1 (0.3)	1.87	1.25	3 (0.9)	1.76	1.20	3 (0.9)	45 (11.4)	1 (0.3)
13	312 (79.4)	16 (4.1)	1 (0.3)	1.87	1.16	2 (0.6)	1.94	1.28	3 (1.0)	1.78	1.18	0 (0.0)	60 (15.2)	0 (0.0)
14	324 (82.4)	11 (2.8)	1 (0.3)	1.88	1.20	4 (1.2)	1.89	1.21	2 (0.6)	1.71	1.08	4 (1.2)	64 (16.2)	0 (0.0)

**Table 2:** Logistic multilevel regression analyses with alcohol use as outcome.

Predictors	Model 1		Model 2		Model 3	
	*OR*	95% *CI*	*OR*	95% *CI*	*OR*	95% *CI*
Level 1 (within-person)						
Daily prejudice events	1.42	[0.79, 2.56]	1.83	[0.90, 3.73]	**1.99**	[1.05, 3.78]
Daily expectations of rejection	1.03	[0.83, 1.27]	1.15	[0.90, 1.46]	0.98	[0.78, 1.22]
Daily concealment	0.87	[0.70, 1.08]	**0.72**	[0.60, 0.86]	0.83	[0.65, 1.05]
Daily internalized stigma	0.93	[0.73, 1.18]	0.93	[0.67, 1.27]	1.00	[0.77, 1.29]
Day of study	1.01	[0.99, 1.03]	1.01	[0.99, 1.03]	1.01	[0.99, 1.03]
Weekend day (1 = Weekend day)	**4.15**	[3.37, 5.12]	**4.16**	[3.36, 5.15]	**4.13**	[3.35, 5.10]
Level 2 (between person)						
Intercept	**14.01**	[8.45, 23.24]	**14.20**	[8.60, 23.45]	**13.85**	[8.37, 22.91]
Person level prejudice events	**7.01**	[1.20, 40.89]	**7.13**	[1.20, 42.25]	**6.81**	[1.16, 39.92]
Person level expectations of rejection	1.08	[0.67, 1.74]	1.07	[0.67, 1.71]	1.08	[0.67, 1.74]
Person level concealment	0.93	[0.61, 1.42]	0.95	[0.63, 1.44]	0.94	[0.61, 1.43]
Person level internalized stigma	0.75	[0.48, 1.17]	0.73	[0.47, 1.14]	0.75	[0.48, 1.18]
Sex assigned at birth (1 = Female)	**0.41**	[0.23, 0.71]	**0.39**	[0.22, 0.69]	**0.40**	[0.23, 0.71]
Gender identity (1 = Gender minority)	1.14	[0.58, 2.22]	1.09	[0.56, 2.15]	1.14	[0.59, 2.20]
Sexual identity (1 = Bisexual)	0.82	[0.44, 1.54]	0.85	[0.45, 1.61]	0.82	[0.43, 1.54]
Sexual identity (1 = Queer/Pansexual/Heterosexual/I don't know/Other)	0.97	[0.47, 2.04]	1.07	[0.51, 2.25]	0.98	[0.46, 2.06]
Cross-level interaction						
Daily prejudice events × Sex assigned at birth (1 = Female)			0.52	[0.20, 1.36]		
Daily expectations of rejection × Sex assigned at birth (1 = Female)			0.75	[0.55, 1.03]		
Daily concealment × Sex assigned at birth (1 = Female)			**1.54**	[1.24, 1.92]		
Daily internalized stigma × Sex assigned at birth (1 = Female)			1.02	[0.70, 1.49]		
Cross-level interaction						
Daily prejudice events × Gender identity (1 = Gender minority)					**0.21**	[0.08, 0.58]
Daily expectations of rejection × Gender identity (1 = Gender minority)					1.33	[0.92, 1.93]
Daily concealment × Gender identity (1 = Gender minority)					1.21	[0.79, 1.85]
Daily internalized stigma × Gender identity (1 = Gender minority)					0.78	[0.53, 1.13]
Deviance	3457.31		3445.64		3443.83	
Decrease in deviance (compared with Model 1)			11.67 (*df* = 4) *p* = .02		13.48 (*df* = 4) *p* = .01	

*Note. OR,* odds ratio. *CI,* confidence interval. Bold *ORs* indicate significance at *p* < .05. The results of the simple slope analyses for the cross-level interaction sex assigned at birth × daily concealment. Male: *OR* = 0.72, 95% *CI*: 0.60−0.86. Female: *OR* = 1.11, 95% *CI*: 0.93−1.32. The results of the simple slope analyses for the cross-level interaction gender identity × daily prejudice events. Cisgender: *OR* = 1.99, 95% *CI*: 1.05−3.78. Gender minority: *OR* = 0.42, 95% *CI*: 0.15−1.18.
