# Supplementary material for: Differences by sex and gender in the association between minority stress and alcohol use among sexual and gender minority youth: A daily diary study
Source: Soc Sci Med. Author manuscript; Available in PMC 2022 Jun 4. (PMC7612808; doi:10.1016/j.socscimed.2021.114679)
Supplement: Supplementary Material [file EMS145485-supplement-Supplementary_Material.docx]

| Table S1A  *Logistic Multilevel Regression Analyses with* ***Prejudice Events as Predictor*** *and Alcohol Use as Outcome* | | | | | | |
| --- | --- | --- | --- | --- | --- | --- |
| Predictors | Model 1 | | Model 2 | | Model 3 | |
|  | *OR* | 95% *CI* | *OR* | 95% *CI* | *OR* | 95% *CI* |
| Level 1 (within-person) |  |  |  |  |  |  |
| Daily prejudice events | 1.32 | [0.74, 2.36] | 1.81 | [0.93, 3.53] | 1.78 | [0.97, 3.26] |
| Day of study | 1.01 | [0.99, 1.03] | 1.01 | [0.99, 1.03] | 1.01 | [0.99, 1.03] |
| Weekend day (1 = Weekend day) | **4.04** | [3.32, 4.92] | **4.03** | [3.30, 4.91] | **4.03** | [3.31, 4.90] |
| Level 2 (between person) |  |  |  |  |  |  |
| Intercept | **12.19** | [7.78, 19.10] | **12.18** | [7.79, 19.05] | **12.24** | [7.82, 19.18] |
| Person level prejudice events | 4.09 | [0.90, 18.49] | 4.02 | [0.89, 18.20] | 3.99 | [0.88, 18.18] |
| Sex assigned at birth (1 = Female) | **0.42** | [0.24, 0.72] | **0.41** | [0.24, 0.72] | **0.42** | [0.24, 0.73] |
| Gender identity (1 = Gender minority) | 1.00 | [0.54, 1.87] | 1.02 | [0.55, 1.91] | 1.02 | [0.54, 1.90] |
| Sexual identity (1 = Bisexual) | 0.76 | [0.41, 1.39] | 0.76 | [0.41, 1.39] | 0.76 | [0.41, 1.39] |
| Sexual identity (1 = Queer/ Pansexual/Heterosexual/I don't know/Other) | 0.85 | [0.42, 1.70] | 0.86 | [0.43, 1.72] | 0.85 | [0.42, 1.72] |
| Cross-level interaction |  |  |  |  |  |  |
| Daily prejudice events × |  |  |  |  |  |  |
| Sex assigned at birth (1 = Female) |  |  | 0.48 | [0.19, 1.20] |  |  |
| Cross-level interaction |  |  |  |  |  |  |
| Daily prejudice events × |  |  |  |  |  |  |
| Gender identity (1 = Gender minority) |  |  |  |  | **0.26** | [0.09, 0.76] |
| Deviance | 3472.15 | | 3470.01 | | 3465.29 | |
| Decrease in deviance  (compared with Model 1) |  | | 2.14 (*df*= 1)  *p* = .14 | | 6.86 (*df*=1)  *p* = .01 | |
| *Note;* *OR*, odds ratio. *CI*, confidence interval. Bold *OR*s indicate significance at *p* < .05  The results of the simple slope analyses for the cross-level interaction gender identity × daily prejudice events:  Cisgender: *OR* = 1.78, 95% *CI*: 0.97-3.26;  Gender minority: *OR* = 0.47, 95% CI: 0.15-1.41. | | | | | | |

| Table S1B  *Logistic Multilevel Regression Analyses with* ***Expectations of Rejection as Predictor*** *and Alcohol Use as Outcome* | | | | | | |
| --- | --- | --- | --- | --- | --- | --- |
| Predictors | Model 1 | | Model 2 | | Model 3 | |
|  | *OR* | 95% *CI* | *OR* | 95% *CI* | *OR* | 95% *CI* |
| Level 1 (within-person) |  |  |  |  |  |  |
| Daily expectations of rejection | 1.02 | [0.85, 1.22] | 1.06 | [0.86, 1.30] | 0.98 | [0.81, 1.19] |
| Day of study | 1.01 | [0.99, 1.03] | 1.01 | [0.99, 1.03] | 1.01 | [0.99, 1.03] |
| Weekend day (1 = Weekend day) | **4.06** | [3.32, 4.95] | **4.06** | [3.32, 4.97] | **4.04** | [3.31, 4.94] |
| Level 2 (between person) |  |  |  |  |  |  |
| Intercept | **12.40** | [7.92, 19.43] | **12.50** | [7.97, 19.62] | **12.29** | [7.82, 19.33] |
| Person level expectations of rejection | 1.01 | [0.78, 1.30] | 1.00 | [0.78, 1.30] | 1.01 | [0.78, 1.30] |
| Sex assigned at birth (1 = Female) | **0.42** | [0.24, 0.72] | **0.42** | [0.24, 0.73] | **0.42** | [0.24, 0.73] |
| Gender identity (1 = Gender minority) | 1.12 | [0.60, 2.10] | 1.12 | [0.60, 2.09] | 1.11 | [0.59, 2.07] |
| Sexual identity (1 = Bisexual) | 0.74 | [0.40, 1.37] | 0.75 | [0.41, 1.38] | 0.74 | [0.40, 1.37] |
| Sexual identity (1 = Queer/ Pansexual/Heterosexual/I don't know/Other) | 0.84 | [0.42, 1.67] | 0.85 | [0.42, 1.68] | 0.84 | [0.42, 1.67] |
| Cross-level interaction |  |  |  |  |  |  |
| Daily expectations of rejection × |  |  |  |  |  |  |
| Sex assigned at birth (1 = Female) |  |  | 0.90 | [0.68, 1.20] |  |  |
| Cross-level interaction |  |  |  |  |  |  |
| Daily expectations of rejection × |  |  |  |  |  |  |
| Gender identity (1 = Gender minority) |  |  |  |  | 1.20 | [0.87, 1.67] |
| Deviance | 3476.69 | | 3476.30 | | 3475.19 | |
| Decrease in deviance  (compared with Model 1) |  | | 0.39 (*df*= 1)  *p* = .53 | | 1.11 (*df*= 1)  *p* = .29 | |
| *Note;* *OR*, odds ratio. *CI*, confidence interval. Bold *OR*s indicate significance at *p* < .05 | | | | | | |

| Table S1C  *Logistic Multilevel Regression Analyses with* ***Concealment as Predictor*** *and Alcohol Use as Outcome* | | | | | | |
| --- | --- | --- | --- | --- | --- | --- |
| Predictors | Model 1 | | Model 2 | | Model 3 | |
|  | *OR* | 95% *CI* | *OR* | 95% *CI* | *OR* | 95% *CI* |
| Level 1 (within-person) |  |  |  |  |  |  |
| Daily concealment | 0.88 | [0.72, 1.06] | **0.78** | [0.63, 0.96] | 0.84 | [0.69, 1.03] |
| Day of study | 1.01 | [0.99, 1.03] | 1.01 | [0.98, 1.03] | 1.01 | [0.99, 1.03] |
| Weekend day (1 = Weekend day) | **4.04** | [3.31, 4.94] | **4.06** | [3.32, 4.96] | **4.05** | [3.32, 4.95] |
| Level 2 (between person) |  |  |  |  |  |  |
| Intercept | **11.95** | [7.60, 18.80] | **12.28** | [7.81, 19.31] | **12.00** | [7.63, 18.87] |
| Person level concealment | 0.96 | [0.77, 1.20] | 0.96 | [0.77, 1.20] | 0.96 | [0.77, 1.20] |
| Sex assigned at birth (1 = Female) | **0.41** | [0.24, 0.71] | **0.42** | [0.24, 0.73] | **0.41** | [0.24, 0.71] |
| Gender identity (1 = Gender minority) | 1.14 | [0.61, 2.12] | 1.13 | [0.61, 2.09] | 1.15 | [0.62, 2.15] |
| Sexual identity (1 = Bisexual) | 0.75 | [0.41, 1.38] | 0.76 | [0.41, 1.40] | 0.75 | [0.41, 1.38] |
| Sexual identity (1 = Queer/ Pansexual/Heterosexual/I don't know/Other) | 0.83 | [0.42, 1.66] | 0.87 | [0.44, 1.73] | 0.84 | [0.42, 1.66] |
| Cross-level interaction |  |  |  |  |  |  |
| Daily concealment × |  |  |  |  |  |  |
| Sex assigned at birth (1 = Female) |  |  | **1.31** | [1.01, 1.69] |  |  |
| Cross-level interaction |  |  |  |  |  |  |
| Daily concealment × |  |  |  |  |  |  |
| Gender identity (1 = Gender minority) |  |  |  |  | 1.21 | [0.84, 1.73] |
| Deviance | 3473.96 | | 3469.14 | | 3472.32 | |
| Decrease in deviance  (compared with Model 1) |  | | 4.82 (*df*= 1)  *p* = .03 | | 1.64 (*df*= 1)  *p* = .20 | |
| *Note;* *OR*, odds ratio. *CI*, confidence interval. Bold *OR*s indicate significance at *p* < .05  The results of the simple slope analyses for the cross-level interaction sex assigned at birth × daily concealment:  Male: *OR* = 0.78, 95% *CI*: 0.63-0.96;  Female: *OR* = 1.02, 95% *CI*: 0.79-1.32. | | | | | | |

| Table S1D  *Logistic Multilevel Regression Analyses with* ***Internalized Stigma as Predictor*** *and Alcohol Use as Outcome* | | | | | | |
| --- | --- | --- | --- | --- | --- | --- |
| Predictors | Model 1 | | Model 2 | | Model 3 | |
|  | *OR* | 95% *CI* | *OR* | 95% *CI* | *OR* | 95% *CI* |
| Level 1 (within-person) |  |  |  |  |  |  |
| Daily internalized stigma | 0.92 | [0.74, 1.15] | 0.92 | [0.70, 1.22] | 0.98 | [0.77, 1.24] |
| Day of study | 1.01 | [0.99, 1.03] | 1.01 | [0.99, 1.03] | 1.01 | [0.99, 1.03] |
| Weekend day (1 = Weekend day) | **4.01** | [3.29, 4.87] | **4.01** | [3.29, 4.87] | **4.01** | [3.30, 4.86] |
| Level 2 (between person) |  |  |  |  |  |  |
| Intercept | **12.95** | [8.11, 20.69] | **12.95** | [8.11, 20.69] | **12.87** | [8.07, 20.52] |
| Person level internalized stigma | 0.84 | [0.62, 1.15] | 0.84 | [0.62, 1.15] | 0.85 | [0.62, 1.15] |
| Sex assigned at birth (1 = Female) | **0.41** | [0.24, 0.71] | **0.41** | [0.24, 0.71] | **0.41** | [0.24, 0.71] |
| Gender identity (1 = Gender minority) | 1.23 | [0.65, 2.32] | 1.23 | [0.65, 2.33] | 1.21 | [0.64, 2.29] |
| Sexual identity (1 = Bisexual) | 0.78 | [0.42, 1.43] | 0.78 | [0.42, 1.43] | 0.78 | [0.42, 1.43] |
| Sexual identity (1 = Queer/ Pansexual/Heterosexual/I don't know/Other) | 0.91 | [0.46, 1.81] | 0.91 | [0.46, 1.81] | 0.91 | [0.46, 1.81] |
| Cross-level interaction |  |  |  |  |  |  |
| Daily internalized stigma × |  |  |  |  |  |  |
| Sex assigned at birth (1 = Female) |  |  | 0.99 | [0.71, 1.39] |  |  |
| Cross-level interaction |  |  |  |  |  |  |
| Daily internalized stigma × |  |  |  |  |  |  |
| Gender identity (1 = Gender minority) |  |  |  |  | 0.82 | [0.58, 1.17] |
| Deviance | 3474.59 | | 3474.59 | | 3473.34 | |
| Decrease in deviance  (compared with Model 1) |  | | 0 (*df*= 1)  *p* = .99 | | 1.25 (*df*= 1)  *p* = .26 | |
| *Note;* *OR*, odds ratio. *CI*, confidence interval. Bold *OR*s indicate significance at *p* < .05 | | | | | | |

| Table S2 Responses from Day 1 Omitted  *Logistic Multilevel Regression Analyses with Alcohol Use as Outcome with Responses from Day 1 Omitted* | | | | | | |
| --- | --- | --- | --- | --- | --- | --- |
| Predictors | Model 1 | | Model 2 | | Model 3 | |
|  | *OR* | 95% *CI* | *OR* | 95% *CI* | *OR* | 95% *CI* |
| Level 1 (within-person) |  |  |  |  |  |  |
| Daily prejudice events | 1.10 | [0.56, 2.15] | 1.46 | [0.66, 3.20] | 1.59 | [0.83, 3.05] |
| Daily expectations of rejection | 1.07 | [0.87, 1.32] | 1.19 | [0.92, 1.54] | 1.02 | [0.85, 1.22] |
| Daily concealment | 0.80 | [0.64, 1.01] | **0.67** | [0.52, 0.86] | **0.79** | [0.62, 0.99] |
| Daily internalized stigma | 0.96 | [0.75, 1.22] | 0.88 | [0.63, 1.25] | 1.03 | [0.80, 1.33] |
| Day of study | 1.01 | [0.98, 1.04] | 1.01 | [0.98, 1.04] | 1.01 | [0.98, 1.04] |
| Weekend day (1 = Weekend day) | **4.38** | [3.49, 5.48] | **4.40** | [3.50, 5.53] | **4.32** | [3.45, 5.42] |
| Level 2 (between person) |  |  |  |  |  |  |
| Intercept | **12.79** | [7.61, 21.51] | **13.49** | [7.96, 22.86] | **12.26** | [7.35, 20.44] |
| Person level prejudice events | **9.51** | [1.15, 78.33] | **10.20** | [1.20, 86.69] | **9.11** | [1.11, 74.45] |
| Person level expectations of rejection | 1.15 | [0.68, 1.95] | 1.14 | [0.67, 1.95] | 1.15 | [0.68, 1.94] |
| Person level concealment | 0.77 | [0.50, 1.37] | 0.77 | [0.49, 1.21] | 0.78 | [0.50, 1.23] |
| Person level internalized stigma | 0.88 | [0.57, 1.37] | 0.87 | [0.56, 1.36] | 0.89 | [0.57, 1.38] |
| Sex assigned at birth (1 = Female) | **0.35** | [0.19, 0.63] | **0.36** | [0.20, 0.65] | **0.34** | [0.19, 0.62] |
| Gender identity (1 = Gender minority) | 1.02 | [0.51, 2.04] | 0.97 | [0.48, 1.97] | 0.97 | [0.49, 1.94] |
| Sexual identity (1 = Bisexual) | 0.77 | [0.41, 1.48] | 0.79 | [0.41, 1.54] | 0.76 | [0.40, 1.46] |
| Sexual identity (1 = Queer/ Pansexual/Heterosexual/I don't know/Other) | 0.95 | [0.44, 2.02] | 1.03 | [0.47, 2.22] | 0.95 | [0.44, 2.04] |
| Cross-level interaction |  |  |  |  |  |  |
| Daily prejudice events × |  |  |  |  |  |  |
| Sex assigned at birth (1 = Female) |  |  | 0.59 | [0.20, 1.75] |  |  |
| Daily expectations of rejection × |  |  |  |  |  |  |
| Sex assigned at birth (1 = Female) |  |  | 0.77 | [0.55, 1.08] |  |  |
| Daily concealment × |  |  |  |  |  |  |
| Sex assigned at birth (1 = Female) |  |  | **1.56** | [1.15, 2.12] |  |  |
| Daily internalized stigma × |  |  |  |  |  |  |
| Sex assigned at birth (1 = Female) |  |  | 1.11 | [0.74, 1.66] |  |  |
| Cross-level interaction |  |  |  |  |  |  |
| Daily prejudice events × |  |  |  |  |  |  |
| Gender identity (1 = Gender minority) |  |  |  |  | **0.23** | [0.08, 0.68] |
| Daily expectations of rejection × |  |  |  |  |  |  |
| Gender identity (1 = Gender minority) |  |  |  |  | **1.46** | [1.01,2.12] |
| Daily concealment × |  |  |  |  |  |  |
| Gender identity (1 = Gender minority) |  |  |  |  | 1.15 | [0.74, 1.80] |
| Daily internalized stigma × |  |  |  |  |  |  |
| Gender identity (1 = Gender minority) |  |  |  |  | 0.72 | [0.49, 1.08] |
| Deviance | 3133.35 | | 3124.59 | | 3120.70 | |
| Decrease in deviance  (compared with Model 1) |  | | 8.76 (*df*=4)  *p* = .07 | | 12.65 (*df*=4)  *p* = .01 | |
| *Note;* *OR*, odds ratio. *CI*, confidence interval. Bold *OR*s indicate significance at *p* < .05  The results of the simple slope analyses for the cross-level interaction sex assigned at birth × daily concealment:  Male: *OR* = 0.67, 95% *CI*: 0.52-0.86;  Female: *OR* = 1.05, 95% *CI*: 0.78-1.40.  The results of the simple slope analyses for the cross-level interaction gender identity × daily prejudice events:  Cisgender : *OR* = 1.59, 95% *CI*: 0.83-3.05;  Gender minority : *OR* = 0.36, 95% *CI*: 0.12-1.08.  The results of the simple slope analyses for the cross-level interaction gender identity × daily expectations of rejection:  Cisgender: *OR* = 1.02, 95% *CI*: 0.85-1.22;  Gender minority: *OR* = 1.48, 95% *CI*: 1.03-2.13. | | | | | | |

Cross lagged models were estimated in Mplus version 8.3 (Muthén & Muthén, 1998-2017) using a WLSMV estimator. Both autoregressive and lagged effects were constrained to be equal because this yielded a more parsimonious model and because constrained models showed a slightly better fit than models in which autoregressive and lagged effects were all freed. Autoregressive paths between day X and day X + 1, day X + 2, and day X + 3 were estimated as this improved the model fit. Both models for the full sample as well as multi-group models for sex assigned at birth and gender identity were estimated. In the multi-group models, only the lagged effect from a daily minority stressors to alcohol use were allowed to differ per group.

| Table S3A  *Cross-Lagged Analyses with* ***Prejudice Events as Predictor*** *and Alcohol Use* | | | | | | | | | | | | | | | | | | |
| --- | --- | --- | --- | --- | --- | --- | --- | --- | --- | --- | --- | --- | --- | --- | --- | --- | --- | --- |
|  | Cross-lagged effects | | | | Stability effects | | | | | | | | | | | | Correlation | |
|  | P day x 🡪 A day x +1 | | A day x🡪 P day x +1 | | P day x 🡪 P day x + 1 | | P day x 🡪 P day x + 2 | | P day x 🡪 P day x + 3 | | A day x 🡪 A day x + 1 | | A day x 🡪 A day x + 2 | | A day x 🡪 A day x + 3 | | P day x 🡪 A day x | |
|  | b | se | b | se | b | se | b | se | b | se | b | se | b | se | b | se | r | se |
| Complete sample | -0.02 | 0.03 | 0.01 | 0.02 | **0.34** | 0.06 | -0.01 | 0.06 | 0.34 | 0.06 | **0.59** | 0.04 | **-0.24** | -0.05 | **0.58** | 0.04 | 0.05 | 0.06 |
|  |  |  |  |  |  |  |  |  |  |  |  |  |  |  |  |  |  |  |
| Multigroup model sex assigned at birth |  |  |  |  |  |  |  |  |  |  |  |  |  |  |  |  |  |  |
| Assigned male at birth | -0.04 | 0.04 | -0.04 | 0.02 | **0.56** | 0.05 | -**0.29** | 0.06 | **0.59** | 0.05 | **0.53** | 0.04 | **-0.16** | 0.05 | **0.60** | 0.04 | 0.00 | 0.01 |
| Assigned female at birth | -0.02 | 0.03 | -0.04 | 0.02 | **0.56** | 0.05 | -**0.29** | 0.06 | **0.59** | 0.05 | **0.53** | 0.04 | **-0.16** | 0.05 | **0.60** | 0.04 | 0.00 | 0.01 |
|  |  |  |  |  |  |  |  |  |  |  |  |  |  |  |  |  |  |  |
| Multigroup model gender identity |  |  |  |  |  |  |  |  |  |  |  |  |  |  |  |  |  |  |
| Cisgender | -0.03 | 0.04 | 0.01 | 0.02 | **0.41** | 0.06 | -0.07 | 0.07 | **0.49** | 0.06 | **0.60** | 0.04 | **-0.27** | 0.05 | **0.68** | 0.04 | 0.10 | 0.06 |
| Gender minority | 0.00 | 0.07 | 0.01 | 0.02 | **0.41** | 0.06 | -0.07 | 0.07 | **0.49** | 0.06 | **0.60** | 0.04 | **-0.27** | 0.05 | **0.68** | 0.04 | 0.10 | 0.06 |
| *Note;* P, prejudice events. A, Alcohol use. Bold estimates indicate significance at *p* <.05  Complete sample: RMSEA = .04. CFI = .83. SRMS = .18.  Multigroup model sex assigned at birth: RMSEA = .04. CFI = .75. SRMS = .20.  Multigroup model gender identity: RMSEA = .04. CFI = .80. SRMS = .20. | | | | | | | | | | | | | | | | | | |

| Table S3B  *Cross-Lagged Analyses with* ***Expected Rejection as Predictor*** *and Alcohol Use* | | | | | | | | | | | | | | | | | | |
| --- | --- | --- | --- | --- | --- | --- | --- | --- | --- | --- | --- | --- | --- | --- | --- | --- | --- | --- |
|  | Cross-lagged effects | | | | Stability effects | | | | | | | | | | | | Correlation | |
|  | E day x 🡪 A day x +1 | | A day x🡪 E day x +1 | | E day x 🡪 E day x + 1 | | E day x 🡪 E day x + 2 | | E day x 🡪 E day x + 3 | | A day x 🡪 A day x + 1 | | A day x 🡪 A day x + 2 | | A day x 🡪 A day x + 3 | | E day x 🡪 A day x | |
|  | b | se | b | se | b | se | b | se | b | se | b | se | b | se | b | se | r | se |
| Complete sample | -0.02 | 0.02 | -0.01 | 0.01 | **0.30** | 0.03 | **0.29** | 0.03 | **0.34** | 0.03 | **0.58** | 0.04 | **-0.24** | 0.05 | **0.65** | 0.04 | 0.03 | 0.03 |
|  |  |  |  |  |  |  |  |  |  |  |  |  |  |  |  |  |  |  |
| Multigroup model sex assigned at birth |  |  |  |  |  |  |  |  |  |  |  |  |  |  |  |  |  |  |
| Assigned male at birth | -0.07 | 0.04 | -0.01 | 0.01 | **0.61** | 0.03 | **0.11** | 0.03 | **0.23** | 0.03 | **0.54** | 0.04 | **-0.18** | 0.05 | **0.62** | 0.04 | 0.03 | 0.03 |
| Assigned female at birth | -0.00 | 0.03 | -0.01 | 0.01 | **0.61** | 0.03 | **0.11** | 0.03 | **0.23** | 0.03 | **0.54** | 0.04 | **-0.18** | 0.05 | **0.62** | 0.04 | 0.03 | 0.03 |
|  |  |  |  |  |  |  |  |  |  |  |  |  |  |  |  |  |  |  |
| Multigroup model gender identity |  |  |  |  |  |  |  |  |  |  |  |  |  |  |  |  |  |  |
| Cisgender | -0.02 | 0.03 | -0.00 | 0.01 | **0.42** | 0.03 | **0.20** | 0.03 | **0.31** | 0.03 | **0.60** | 0.04 | **-0.27** | 0.05 | **0.67** | 0.04 | 0.02 | 0.03 |
| Gender minority | 0.02 | 0.04 | -0.00 | 0.01 | **0.42** | 0.03 | **0.20** | 0.03 | **0.31** | 0.03 | **0.60** | 0.04 | **-0.27** | 0.05 | **0.67** | 0.04 | 0.02 | 0.03 |
| *Note;* E, expected rejection. A, Alcohol use. Bold estimates indicate significance at *p* <.05  Complete sample: RMSEA = .04. CFI = .86. SRMS = .13.  Multigroup model sex assigned at birth: RMSEA = .03. CFI = .91. SRMS = .15.  Multigroup model gender identity: RMSEA = .04. CFI = .87. SRMS = .16. | | | | | | | | | | | | | | | | | | |

| Table S3C  *Cross-Lagged Analyses with* ***Concealment as Predictor*** *and Alcohol Use* | | | | | | | | | | | | | | | | | | |
| --- | --- | --- | --- | --- | --- | --- | --- | --- | --- | --- | --- | --- | --- | --- | --- | --- | --- | --- |
|  | Cross-lagged effects | | | | Stability effects | | | | | | | | | | | | Correlation | |
|  | C day x 🡪 A day x +1 | | A day x🡪 C day x +1 | | C day x 🡪 C day x + 1 | | C day x 🡪 C day x + 2 | | C day x 🡪 C day x + 3 | | A day x 🡪 A day x + 1 | | A day x 🡪 A day x + 2 | | A day x 🡪 A day x + 3 | | C day x 🡪 A day x | |
|  | b | se | b | se | b | se | b | se | b | se | b | se | b | se | b | se | r | se |
| Complete sample | -0.01 | 0.02 | 0.00 | 0.01 | **0.44** | 0.05 | **0.48** | 0.04 | 0.02 | 0.06 | **0.57** | 0.04 | **-0.24** | 0.05 | **0.65** | 0.04 | -0.04 | 0.03 |
|  |  |  |  |  |  |  |  |  |  |  |  |  |  |  |  |  |  |  |
| Multigroup model sex assigned at birth |  |  |  |  |  |  |  |  |  |  |  |  |  |  |  |  |  |  |
| Assigned male at birth | -0.02 | 0.03 | 0.01 | 0.01 | **0.56** | 0.05 | **0.42** | 0.05 | -0.03 | 0.07 | **0.54** | 0.04 | **-0.17** | 0.02 | **0.62** | 0.04 | -0.05 | 0.03 |
| Assigned female at birth | 0.00 | 0.02 | 0.01 | 0.01 | **0.56** | 0.05 | **0.42** | 0.05 | -0.03 | 0.07 | **0.54** | 0.04 | **-0.17** | 0.02 | **0.62** | 0.04 | -0.05 | 0.03 |
|  |  |  |  |  |  |  |  |  |  |  |  |  |  |  |  |  |  |  |
| Multigroup model gender identity |  |  |  |  |  |  |  |  |  |  |  |  |  |  |  |  |  |  |
| Cisgender | -0.01 | 0.02 | 0.01 | 0.01 | **0.59** | 0.05 | **0.38** | 0.05 | -0.01 | 0.07 | **0.58** | 0.04 | **-0.26** | 0.05 | **0.68** | 0.04 | -0.06 | 0.03 |
| Gender minority | 0.02 | 0.04 | 0.01 | 0.01 | **0.59** | 0.05 | **0.38** | 0.05 | -0.01 | 0.07 | **0.58** | 0.04 | **-0.26** | 0.05 | **0.68** | 0.04 | -0.06 | 0.03 |
| *Note;* C, concealment. A, Alcohol use. Bold estimates indicate significance at *p* <.05  Complete sample: RMSEA = .04. CFI = .86. SRMS = .14.  Multigroup model sex assigned at birth: RMSEA = .03. CFI = .91. SRMS = .15.  Multigroup model gender identity: RMSEA = .03. CFI = .89. SRMS = .16. | | | | | | | | | | | | | | | | | | |

| Table S3D  *Cross-Lagged Analyses with* ***Internalized Stigma*** *and Alcohol Use* | | | | | | | | | | | | | | | | | | |
| --- | --- | --- | --- | --- | --- | --- | --- | --- | --- | --- | --- | --- | --- | --- | --- | --- | --- | --- |
|  | Cross-lagged effects | | | | Stability effects | | | | | | | | | | | | Correlation | |
|  | I day x 🡪 A day x +1 | | A day x🡪 I day x +1 | | I day x 🡪 I day x + 1 | | I day x 🡪 I day x + 2 | | I day x 🡪 I day x + 3 | | A day x 🡪 A day x + 1 | | A day x 🡪 A day x + 2 | | A day x 🡪 A day x + 3 | | I day x 🡪 A day x | |
|  | b | se | b | se | b | se | b | se | b | se | b | se | b | se | b | se | r | se |
| Complete sample | **-0.04** | 0.02 | -0.01 | 0.01 | **0.45** | 0.04 | **0.19** | 0.04 | **0.31** | 0.03 | **0.59** | 0.04 | **-0.26** | 0.05 | **0.65** | 0.04 | 0.06 | 0.03 |
|  |  |  |  |  |  |  |  |  |  |  |  |  |  |  |  |  |  |  |
| Multigroup model sex assigned at birth |  |  |  |  |  |  |  |  |  |  |  |  |  |  |  |  |  |  |
| Assigned male at birth | -0.06 | 0.04 | -0.01 | 0.01 | **0.51** | 0.04 | **0.18** | 0.03 | **0.26** | 0.03 | **0.54** | 0.04 | **-0.18** | 0.05 | **0.62** | 0.04 | 0.04 | 0.03 |
| Assigned female at birth | -0.02 | 0.02 | -0.01 | 0.01 | **0.51** | 0.04 | **0.18** | 0.03 | **0.26** | 0.03 | **0.54** | 0.04 | **-0.18** | 0.05 | **0.62** | 0.04 | 0.04 | 0.03 |
|  |  |  |  |  |  |  |  |  |  |  |  |  |  |  |  |  |  |  |
| Multigroup model gender identity |  |  |  |  |  |  |  |  |  |  |  |  |  |  |  |  |  |  |
| Cisgender | **-0.06** | 0.02 | -0.01 | 0.01 | **0.55** | 0.04 | **0.14** | 0.03 | **0.27** | 0.03 | **0.60** | 0.04 | **-0.26** | 0.05 | **0.66** | 0.04 | 0.06 | 0.03 |
| Gender minority | 0.01 | 0.04 | -0.01 | 0.01 | **0.55** | 0.04 | **0.14** | 0.03 | **0.27** | 0.03 | **0.60** | 0.04 | **-0.26** | 0.05 | **0.66** | 0.04 | 0.06 | 0.03 |
| *Note;* I, internalized stigma. A, Alcohol use. Bold estimates indicate significance at *p* <.05  Complete sample: RMSEA = .04. CFI = .86. SRMS = .14.  Multigroup model sex assigned at birth: RMSEA = .03. CFI = .90. SRMS = .16.  Multigroup model gender identity: RMSEA = .03. CFI = .89. SRMS = .15. | | | | | | | | | | | | | | | | | | |
